# Where your heart lies across the Atlantic may demand further assessment in cardiovascular management for non-cardiac surgery

**DOI:** 10.1093/ehjopen/oeae105

**Published:** 2024-12-17

**Authors:** Benjamin Marchandot, Adrien Carmona, Olivier Morel

**Affiliations:** Division of Cardiovascular Medicine, Strasbourg University Hospital, 67000 Strasbourg, France; Research Unit-UR3074, Translational Cardiovascular Medicine, University of Strasbourg, 67000 Strasbourg, France; Division of Cardiovascular Medicine, Strasbourg University Hospital, 67000 Strasbourg, France; Research Unit-UR3074, Translational Cardiovascular Medicine, University of Strasbourg, 67000 Strasbourg, France; Division of Cardiovascular Medicine, Strasbourg University Hospital, 67000 Strasbourg, France; Research Unit-UR3074, Translational Cardiovascular Medicine, University of Strasbourg, 67000 Strasbourg, France; Hanoï Medical University, Hanoi, Vietnam

**Keywords:** Non-cardiac surgery, Guidelines, Cardiac Biomarkers

Let us get straight to the point: assessing the perioperative cardiac risk for a 65-year-old man without cardiovascular (CV) risk factors or history of CV disease, scheduled for an elective cholecystectomy, appears simple. Or does it?

According to the recent guidelines released by the European Society of Cardiology (ESC) in 2022,^1^ cholecystectomy holds an intermediate surgical risk (1–5%), and according to the pathway proposed by the ESC recommendations, our 65-year-old patient requires an electrocardiogram testing, biomarkers monitoring [hs-cTn T/I (Class I)] and/or [B-type natriuretic peptide (BNP)/NT-proBNP (Class IIa)], and an additional functional capacity assessment (Class IIa). Across the Atlantic, the 2024 ACC/AHA guidelines^2^ advocate employing risk-prediction tools (level of evidence: 2a) to forecast the likelihood of perioperative major cardiac events (MACE) and prompt additional examinations. Consequently, the revised cardiac risk index (RCRI),^3^ American College of Surgeons (ACS) NSQIP MICA,^4^ and ACS NSQIP Surgical Risk Calculator^5^ are endorsed for a comprehensive risk assessment. RCRI is 1; ACS NSQIP is at a lower-than-average risk, and the Gupta perioperative cardiac risk is 0.04. All these scores suggest a low risk of MACE, indicating that no further testing is necessary according to the pre-operative assessment scheme outlined in the US guidelines. When crossing the US-Canada border, our patient possesses an RCRI score of ≥1 and is aged 65 or older. The 2016 Canadian guidelines^6^ recommend the measurement of natriuretic peptide to inform subsequent testing decisions (*Figure 1*).

**Figure 1:**
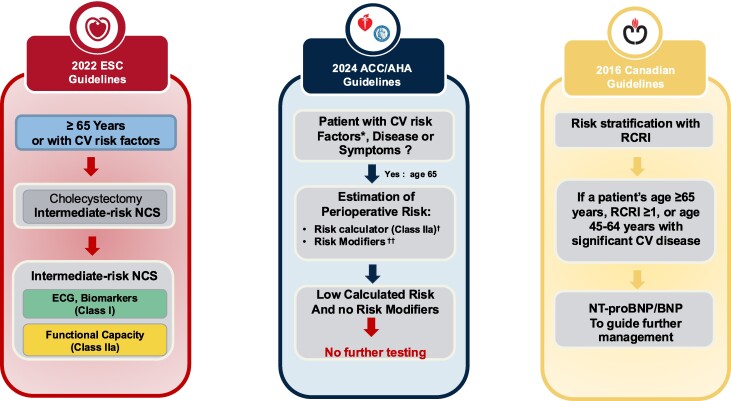
Variations among the United States, ESC, and Canadian guidelines in evaluating perioperative cardiac risk for a 65-year-old male with no cardiovascular risk factors or history of cardiovascular disease, and scheduled for an elective cholecystectomy. BNP, B-type natriuretic peptide; CAD, coronary artery disease; ECG, electrocardiogram; MACE, major cardiac events; NCS, non-cardiac surgery; RCRI, revised cardiac risk index. *Cardiovascular risk factors: hypertension, smoking, high cholesterol, diabetes, women age >65 years; men age >55 years; obesity; family history of premature coronary artery disease. †Determining elevated calculated risk depends on the calculator used. Traditionally, revised cardiac risk index >1 or a calculated risk of major cardiac events with any perioperative risk calculator >1% is used as a threshold to identify patients at elevated risk. †† Determining if the patient has any of the following risk modifiers: severe valvular heart disease; severe pulmonary hypertension, elevated risk congenital heart disease, priori coronary stents/coronary artery bypass grafting; recent stroke; cardiac implantable electronic device (pacemaker/implantable cardioverter defibrillators); frailty.

When it comes to intermediate-risk surgical procedures and patients aged 65 and older without CV factors or a history of CV disease, the ESC guidelines come with a Class I recommendation for troponin assessment and Class IIa for BNP/NT-proBNP measurement, independent of the clinician’s presumption on HF. In the context of elevated-risk non-cardiac surgery (NCS) only, the American approach prioritizes an initial assessment of the patient’s functional capacity. If functional capacity is poor (METS <4) or unknown, the guidelines recommend asking the clinician, ‘Will additional biomarker testing, including BNP/NT-proBNP (Class IIa) and cardiac troponin (Class IIb), affect decision-making and/or perioperative care?’ The difference between the ESC and ACC/AHA recommendations may appear subtle, but it is clinically significant, as it guides whether to proceed or not with biomarker monitoring.

The Canadian guidelines tend to offer an interesting perspective. Indeed, these Canadian recommendations, published in 2016, find an echo in the VISION NT-proBNP study, published 4 years later in 2020.^7^ Pre-operative NT-proBNP measurements from 10 402 patients showed that compared with pre-operative NT-proBNP <100 ng/L (the reference group), values of ≥100 to <200, ≥200 to <1500, and ≥1500 were associated with adjusted HRs of 2.27 (95% CI 1.90–2.70), 3.63 (95% CI 3.13–4.21), and 5.82 (95% CI 4.81–7.05), and corresponding incidences of the composite of vascular death and myocardial injury (12.3, 20.8, and 37.5%, respectively). Pre-operative NT-proBNP improved risk prediction and clinical stratification by RCRI, resulting in a net absolute reclassification improvement of 258 per 1000 patients. A study published by Gualandro *et al*.^8^ in 2023 reported a 2.5% incidence of post-operative acute heart failure (HF) in 9164 consecutive high-risk patients undergoing NCS. Acute HF occurred most likely on post-operative Day 2 and equally in patients with and without known chronic HF. Significant predictors were chronic HF, urgent or emergency procedures, and diabetes mellitus. In a derived cohort, Meister *et al*.^9^ showed that pre-operative cardiac troponin improved risk prediction discrimination for perioperative myocardial infarction/injury beyond a clinical risk index. The analyses in both studies focus on high-risk patients aged ≥65 years or ≥45 years with a history of CAD, peripheral artery disease, or stroke, who are either undergoing major NCS or scheduled for arterial vascular surgery.

Pre-operative CV risk prediction rests upon four essential pillars: (i) clinical risk indices, (ii) functional capacity assessment, (iii) non-invasive imaging techniques, and (iv) cardiac biomarkers. Devereaux *et al*.^10^ nicely addressed the strengths and limitations of each. Clinical risk indices, while widely utilized, show limited discriminatory power and propensity for underestimating risk. Notably, and somewhat unexpectedly, the 2022 ESC recommendations still propose that risk calculators may be used in addition to, or even as an alternative to, the evaluation of surgery-related and patient-related risk factors. Functional capacity assessment shows modest predictive capabilities in risk stratification. The accessibility and cost associated with non-invasive imaging pose significant challenges, restricting their widespread use. Finally, cardiac biomarkers offer cost-effective and convenient solutions with substantial predictive value.

Why are we raising a debate and questioning the pathway to follow in the very specific subgroup of patients aged 65 and older with intermediate surgical risk, no CV risk factors, and no CV disease? On the one hand, early detection is a prerequisite for early treatment and improved outcomes. Given the unacceptably high morbidity and mortality in patients developing MACE after NCS, strategies aimed at improving outcomes are more than welcome. On the other hand, cost-effectiveness strategies and resource allocation for healthcare systems cannot simply be disregarded. The 2022 ESC document states that by 2030, one-fifth of individuals aged over 75 years will undergo surgery each year. While individuals aged 75 and older have a greater risk of perioperative MACEs compared with their younger counterparts (9.5% vs. 4.8%, *P* < 0.001),^11^ age *per se* poses a smaller risk compared with emergency surgery or comorbid conditions such as pulmonary and renal disease. Seventy-five is the new 65, both at the med corner and more broadly in life.

The 2022 ESC guidelines strongly endorse the liberal use of perioperative biomarker testing, recommending its application prior to intermediate- and high-risk NCS, as well as at 24 and 48 h post-operatively. In contrast, the 2024 ACC/AHA guidelines primarily emphasize pre-operative biomarker management. The key distinction in biomarker testing between the 2022 ESC recommendations and the 2024 ACC/AHA guidelines lies in the strength of evidence supporting the use of cardiac troponin. The 2022 ESC guidelines assign a Class 1 recommendation for pre-operative troponin measurement in patients with known CVD, CV risk factors (including age ≥65 years), or symptoms suggestive of CVD undergoing intermediate- or high-risk NCS. In contrast, the 2024 ACC/AHA guidelines provide a Class IIa recommendation for pre-operative troponin measurement in patients with known CVD, or age ≥65 years, or age ≥45 years with symptoms indicative of CVD undergoing elevated-risk NCS.

A recent position paper from the European Society of Anaesthesiology and Intensive Care (ESAIC) has garnered attention.^12^ Particularly noteworthy are the recommendations regarding pre-operative, post-operative, and combined cardiac troponin measurements for prognosis and risk prediction, which appear weaker in the ESAIC position paper compared with the ESC 2022 guidelines. For instance, routine pre-operative assessment of cardiac troponins is suggested to aid in evaluating the risk of adverse outcomes before non-cardiac surgeries. However, this recommendation is weak due to very low-quality evidence. Additionally, the routine inclusion of pre-operative cardiac troponins for predicting post-operative events lacks a specific endorsement from this group. The panel determined that crucial aspects concerning the utilization of combined pre- and post-operative cardiac troponin measurements remain largely uncertain. In a diplomatic gesture, the authors acknowledge that despite differences between the ESAIC and 2022 ESC recommendations, they may still align in direction and are not necessarily incompatible.

In the 2022 ESC paper, recommendations for pre-operative risk assessment, electrocardiography, and biomarkers are supported by either a Class C or B level of evidence. In the 2024 ACC/AHA guidelines, the recommendations for pre-operative biomarker testing are supported by a level of evidence B-NR, indicating moderate-quality evidence derived from non-randomized (NR) studies. This underscores the need for further research, as multiple randomized clinical trials or meta-analyses are still required to strengthen the certainty of these recommendations. This sentiment is echoed by the recent ESAIC initiative, which employs the GRADE methodology to evaluate the level of evidence for biomarker testing across three key applications: prognosis, risk prediction, and biomarker-based management strategies. The findings from this initiative reveal a generally low level of evidence, with none exceeding a moderate level of confidence.

Getting back our 65-year-old man without CV risk factors or history of CV disease, scheduled for an elective cholecystectomy, the Canadian approach takes the lead in our mind. Pre-operative troponin holds a Class I recommendation in the 2022 ESC guidelines in this scenario. BNP/NT-proBNP holds a Class IIa recommendation and is more likely to reclassify the pre-operative management of our patient.

The ‘perioperative team’—comprising anaesthetists, surgeons, cardiologists, intensivists, geriatricians, and others—should evaluate their practices and perspectives on NCS management in light of these new recommendations. The acceptance, utilization, and applicability of these guidelines by physicians still need to be thoroughly assessed.

## Data Availability

No data were generated or analysed for or in support of this paper.

## Lead author biography

Dr. Benjamin Marchandot is a junior researcher at the Research Unit UR3074 Translational Cardiovascular Medicine, University of Strasbourg, France. He is an active member of GERCA (Groupe pour l’Enseignement et la Recherche Cardiovasculaire en Alsace), a research group dedicated to the study of CV disease, thrombosis, and haemostasis.
